# Metabolomics Network Analysis of Various Genotypes Associated with Schizophrenia Gene Variant

**DOI:** 10.3390/metabo15080551

**Published:** 2025-08-15

**Authors:** Hema Sekhar Reddy Rajula, Cristina Piras, Karolina Krystyna Kopeć, Antonio Noto, Martina Spada, Katia Lilliu, Mirko Manchia, Michele Mussap, Luigi Atzori, Vassilios Fanos

**Affiliations:** 1 Department of Surgical Sciences, University of Cagliari, 09042 Cagliari, Italy; hemasekhar09@gmail.com (H.S.R.R.); k.kopec@studenti.unica.it (K.K.K.); mumike153@gmail.com (M.M.); vafanos@tiscali.it (V.F.); 2 Ph.D. Student Marie Sklodowska-Curie CAPICE Project, Department of Surgical Sciences, University of Cagliari, 09042 Cagliari, Italy; 3 Department of Biomedical Sciences, University of Cagliari, 09042 Cagliari, Italy; cristina.piras@unica.it (C.P.); martina.spada@unica.it (M.S.); kati9513@gmail.com (K.L.); latzori@unica.it (L.A.); 4 Department of Mechanical, Chemical and Materials Engineering, University of Cagliari, 09123 Cagliari, Italy; 5 Section of Psychiatry, Department of Medical Science and Public Health, University of Cagliari, 09125 Cagliari, Italy; mirko.manchia@unica.it; 6 Department of Pharmacology, Dalhousie University, Halifax, NS B3H 4R2, Canada

**Keywords:** schizophrenia, NOS1AP, metabolomics, network analysis, enrichment analysis

## Abstract

Background: This study investigates metabolic perturbations in serum samples associated with different genotypes (AA, AC, and CC) of the schizophrenia susceptibility gene NOS1AP-rs12742393. Methods: Publicly available datasets acquired using ultra-performance liquid chromatography–quadrupole time-of-flight mass spectrometry (UPLC–QTOFMS) were analyzed by employing network and enrichment approaches. Results: Key metabolites, including tryptophan, 2-aminobutyric acid, palmitic acid, and 5-hydroxytryptophan, were strongly linked to metabolic networks in genotypes AA-AC and AA. Enrichment analysis was conducted to identify metabolite sets differentially distributed across these genotypes, with a particular focus on genotype AA. Conclusions: The findings suggest that NOS1AP-rs12742393 contributes to complex metabolic alterations involving amino acids, organic compounds, fatty acids, and cholic acids. Moreover, serum metabolome analysis demonstrates sufficient sensitivity and specificity to provide insights into NOS1AP-rs12742393 genotype-associated metabolic profiles, supporting a network-based approach to understanding schizophrenia susceptibility.

## 1. Introduction

Schizophrenia (SCZ) is a severe chronic psychiatric disorder affecting approximately 0.5–1% of the global population. It is characterized by positive symptoms (hallucinations and delusions), negative symptoms (emotional blunting and apathy), and cognitive impairment [1]. A significant proportion of patients exhibit a suboptimal response or resistance to antipsychotic treatments, which remain the cornerstone of pharmacological therapy [2]. Given the high socio-economic burden of SCZ, substantial research has focused on elucidating its etiology and underlying pathophysiological mechanisms. The latest GWAS of schizophrenia identified common variant associations at 287 distinct genomic loci. These associations were enriched in genes expressed specifically in excitatory and inhibitory neurons of the central nervous system, with minimal involvement of other tissues or cell types. Through integrative fine-mapping and analysis of functional genomic annotations, they prioritize 120 likely causal genes, including 106 that encode proteins. Notably, 16 of these genes harbor credible causal variants in coding or untranslated regions [3].

While polygenic risk scoring has emerged as a promising approach for predicting disease susceptibility, its clinical utility remains limited due to insufficient predictive accuracy. Additionally, genetic data are inherently static and do not capture dynamic biological perturbations, such as those induced by pharmacological treatment. In this context, metabolomics provides a functional readout of phenotype by detecting, identifying, and quantifying metabolites in biological samples such as serum and urine. Metabolites (<1500 Da) represent the downstream products of cellular metabolism, reflecting gene expression, transcript activity, and protein function, thereby offering a unique perspective on small-molecule regulation [4]. Metabolomics holds significant potential for translating metabolic fingerprints into personalized therapeutic strategies and serves as a powerful tool for identifying metabolic pathways and networks implicated in SCZ. As an emerging component of network medicine, metabolomics enables systematic exploration of disease complexity, facilitating the identification of disease modules, pathways, and molecular interconnections among seemingly distinct pathological phenotypes driven by external perturbations [5].

Unlike traditional biochemical approaches that focus on individual metabolites, metabolomics provides a comprehensive quantitative profile of metabolic fluctuations associated with pathological conditions [6]. Network analysis further enhances this approach by modeling complex interactions between biological entities. Scale-free networks, characterized by a few highly connected hubs alongside numerous weakly connected nodes, have been extensively utilized in biological sciences to elucidate key regulatory mechanisms. The application of network-based approaches in disease research has yielded novel insights, advancing our understanding of biological complexity [7].

The NOS1AP gene encodes nitric oxide synthase 1 adaptor protein (NOS1AP), which directly binds to neuronal nitric oxide synthase (nNOS). NOS1AP modulates nitric oxide (NO) release at synaptic sites through mechanisms mediated by the N-methyl-D-aspartate receptor (NMDAR) [8]. NMDAR hypofunction has long been implicated in SCZ pathophysiology, positioning NOS1AP as a strong candidate gene for disease susceptibility [9]. Notably, a variant of NOS1AP (rs12742393) has been linked to SCZ, with the A allele identified as the risk variant [10,11]. However, the molecular mechanisms, metabolic alterations, biological pathways, and networks associated with NOS1AP remain largely unexplored. Metabolomics and network analysis offer a valuable approach for characterizing the endogenous metabolic landscape across cells, biofluids, and tissues, potentially facilitating the identification of biomarkers and pathogenic mechanisms, as well as biochemical and structural similarity relationships underlying SCZ [12,13].

In this study, we employed network and enrichment analysis to characterize the properties of metabolites and their complex biochemical and structural relationships of different NOS1AP-rs12742393 genotypes, utilizing quantified metabolomic data from Zhang et al. [11]. Our findings provide insights into genotype-specific metabolic perturbations and their potential implications in SCZ pathogenesis.

## 2. Materials and Methods

The dataset utilized in this study is available at the NIH Common Fund’s National Metabolomics Data Repository (NMDR) website, the Metabolomics Workbench, https://www.metabolomicsworkbench.org (accessed on 20 March 2024), where it has been assigned Project ID PR000416. The data can be accessed directly via their Project DOI: 10.21228/M88S3F. The dataset from Zhang et al. comprises 147 healthy individuals with normal glucose tolerance, including 49 subjects homozygous for the NOS1AP-rs12742393 AA genotype, 49 heterozygous (AC), and 49 homozygous for the CC genotype [11]. As described by Zhang et al., serum samples were collected following an 8 h fasting period and stored at −80 °C until analysis. Metabolomic profiling was performed using ultra-performance liquid chromatography–quadrupole time-of-flight mass spectrometry (UPLC–QTOFMS) in both electrospray ionization positive (ESI+) and electrospray ionization negative (ESI−) acquisition modes [11].

### 2.1. Statistical Analysis

Both univariate and multivariate statistical analyses were performed. Univariate analysis was used to assess differences in mean metabolite concentrations (log10-transformed) between genotypes of the NOS1AP-rs12742393 variant. Two separate datasets were generated corresponding to positive (ESI+) and negative (ESI−) ionization modes. Orthogonal projections to latent structures discriminant analysis (PLS-DA) were applied using SIMCA software (ver. 15.0, Umetrics, Sweden) [14] to reduce model complexity and enhance sample discrimination. Supervised analysis yielded a set of variables important for projections (VIP) [15].

For multivariate analysis, principal component analysis (PCA) and partial least squares discriminant analysis (PLS-DA) were performed. PCA was employed to assess sample homogeneity within each genotype group (AA, AC, and CC) and to identify potential trends or outliers. PLS-DA was used to construct a predictive model by maximizing covariance between the dataset (X) and class membership (Y) while filtering out non-discriminatory variations.

Model validity was assessed through cumulative modeled variation in the X and Y matrices (R^2^X and R^2^Y, respectively) and cross-validated predictive ability (Q^2^). The robustness of the PLS-DA model was evaluated using 7-fold cross-validation and a permutation test (500 permutations).

To identify key metabolites contributing to class separation, loadings plots were examined. Metabolites with VIP scores >1.0 were considered significant [16]. The VIP method ranked variables based on their contribution to the model, retaining only those exceeding the predefined threshold of 1.0 [16].

### 2.2. Network Analysis Approach

Network analysis was conducted to characterize the properties of metabolites and their complex biochemical and structural relationships. MetaMapR, an R-based statistical programming environment (v3.0.1), was employed to integrate biochemical relationships with structural similarity, mass spectral similarity, and correlation data, treating all enzymatic relationships as undirected [17].

The Data Analysis and Visualization engine (DAVe), a web-based platform, was utilized to process SCZ datasets, where subjects were listed in rows and metabolites in columns [17]. The DAVe network module provided statistical tools to construct various networks, including correlation networks, biochemical relationship networks, and structural similarity networks. Metabolic networks were mapped using DAVe (available at [https://creative-data.science/dave/], last accessed 30 October 2024) in conjunction with KEGG (Kyoto Encyclopedia of Genes and Genomes [18]) and PubChem CID [19] identifiers (NCBI National Center for Biotechnology Information, PubChem compound database, available at [http://pubchem.ncbi.nlm.nih.gov] (accessed on 1 June 2024). The resulting metabolite relationships were represented as nodes (variables) and edges (connections). Structural similarity was assessed using PubChem Substructure Fingerprints (available at [ftp://ftp.ncbi.nlm.nih.gov/pubchem/specifications/pubchem_fingerprints.txt]), last accessed 30 October 2024). Tanimoto coefficients were computed for each metabolite, with a similarity threshold set at ≥0.7; variables scoring ≤0.7 were excluded, which maintains distinct, non-overlapping network modularity across biochemical classes [19]. A more detailed description of the structural similarity threshold selection process is provided elsewhere [20]. The final metabolic networks were visualized using Cytoscape [21].

### 2.3. Enrichment Analysis Approach

Enrichment analysis was performed using MetaboAnalyst 4.0 (www.metaboanalyst.ca (accessed on 15 July 2024), an integrated web-based platform designed for comprehensive metabolomics data analysis and interpretation [22]. Metabolite Set Enrichment Analysis (MSEA) is designed to aid in the identification and interpretation of biologically meaningful patterns in human or mammalian metabolite concentration changes [23]. Central to MSEA is a curated library of approximately 1000 predefined metabolite sets, encompassing a broad range of metabolic pathways, disease phenotypes, biofluids, and tissue-specific profiles. In addition, MSEA supports the use of custom user-defined metabolite sets to enable more specialized or context-specific analyses [23].

Enrichment was conducted by selecting a metabolite set library linked to pathway-associated metabolite sets (KEGG [18], 30 October 2024). Over-Representation Analysis (ORA) was applied when a list of compound names was provided. ORA uses the hypergeometric test to determine whether a specific metabolite set is represented more frequently than expected by chance within the given compound list. One-tailed *p*-values were calculated and adjusted for multiple comparisons.

Enriched pathways were ranked according to their log-transformed *p*-values and pathway impact, as determined by topology analysis. A summary plot for MSEA was generated, ranking pathways by Holm-adjusted *p*-values, which were computed using the Holm–Bonferroni method to address the issue of multiple comparisons, a widely applied technique for large-scale data analysis. Fold enrichment was calculated by dividing the observed number of hits by the expected number of hits. Metabolic pathways were ordered from top to bottom based on decreasing statistical significance, which corresponds to increasing nominal *p*-values. Pathways with the smallest *p*-values (most significant) appear at the top, while those with larger *p*-values are listed lower. Fold enrichment quantifies pathway over-representation and is commonly visualized using color gradients, with red indicating higher fold enrichment [22,23].

## 3. Results

**Figure 1:**
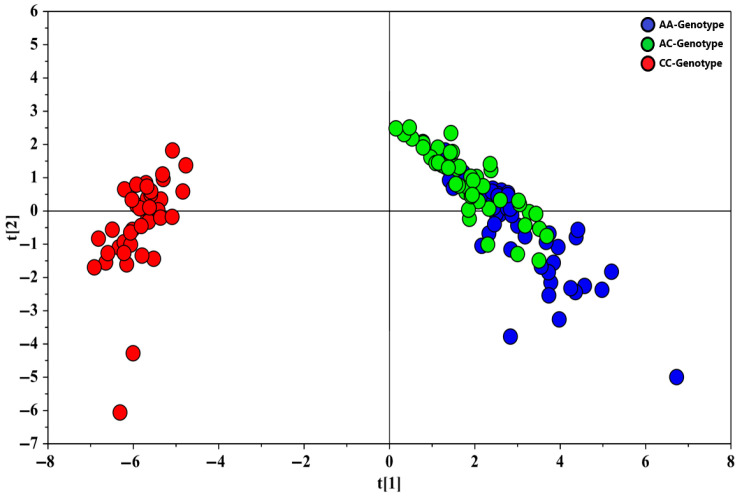
PLS-DA score plot showing the serum metabolic profile (ESI+ acquisition mode) of AA (blue dots), AC (green dots), and CC (red dots) subjects. PLS-DA model was created using two components and described by R^2^X, R^2^Y, and Q^2^ values of 0.581, 0.570, and 0.531, respectively.

The demographic and clinical characteristics of the study population are summarized in Table 1. Among the 147 subjects, the AA, AC, and CC carriers of rs12742393 were well matched with respect to age, sex, and BMI. A principal component analysis (PCA) was first performed on metabolomic profiles identified in both the positive and negative (ESI+ and ESI−) acquisition modes (). The results indicated that the different classes were consistently grouped for both acquisition modes, with no outliers detected. To refine the analysis and focus on metabolic differences relevant to the pathology of interest, partial least squares discriminant analysis (PLS-DA) was applied to the dataset obtained from the positive (ESI+) mode. The PLS-DA score plot revealed no significant metabolic differences between AA and AC genotypes, whereas CC genotype samples were distinct, showing significant differences in metabolomic profiles between the three genotype groups. The validity of the PLS-DA model was assessed through a permutation test (500 iterations), yielding a Q^2^ intercept value of −0.079, confirming the model’s statistical reliability (Figure 1).

A loading plot (was used to identify metabolites contributing to serum metabolome modifications in AA and AC subjects compared to CC subjects. Metabolites with a VIP score greater than 1.0 were considered significant (Table 2).

**Figure 2:**
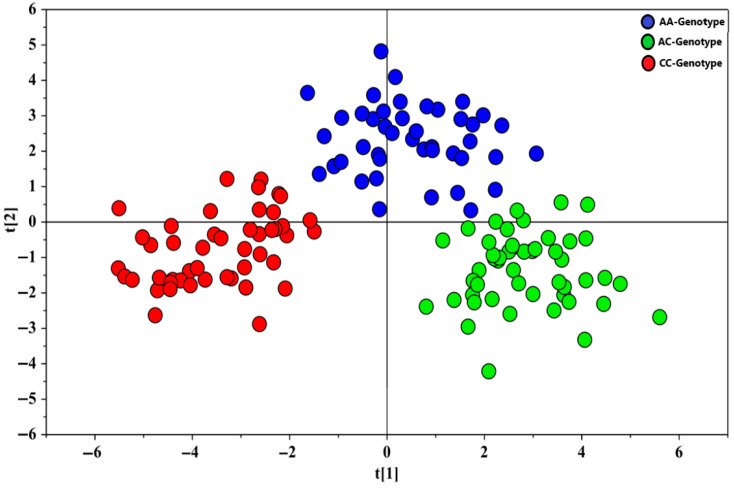
PLS-DA score plot showing the serum metabolic profile (ESI- acquisition mode) of AA (blue dots), AC (green dots), and CC (red dots) subjects. PLS-DA model was performed using two components and described by R^2^X, R^2^Y, and Q^2^ values of 0.250, 0.677, and 0.596, respectively.

Similarly, for the negative (ESI−) acquisition mode, PLS-DA was conducted on the dataset obtained from this mode. The PLS-DA score plot (Figure 2) indicated clear separation of the serum samples into three distinct classes based on genotype: AA, AC, and CC. The validity of the PLS-DA model was confirmed by a permutation test (500 iterations), which returned a Q^2^ intercept value of −0.227, supporting the robustness of the model.

A loading plot was again used to identify the metabolites contributing to serum metabolome modifications across AA, AC, and CC genotypes. Only metabolites with VIP > 1 were considered (Table 3). The serum metabolomic profiles of different NOS1AP genotypes revealed a total of 30 metabolites associated with the AA-AC genotypes and 126 metabolites associated with the AA genotype, identified from the positive and negative (ESI+ and ESI−) modes, respectively.

### 3.1. Network Analysis Results

**Figure 3:**
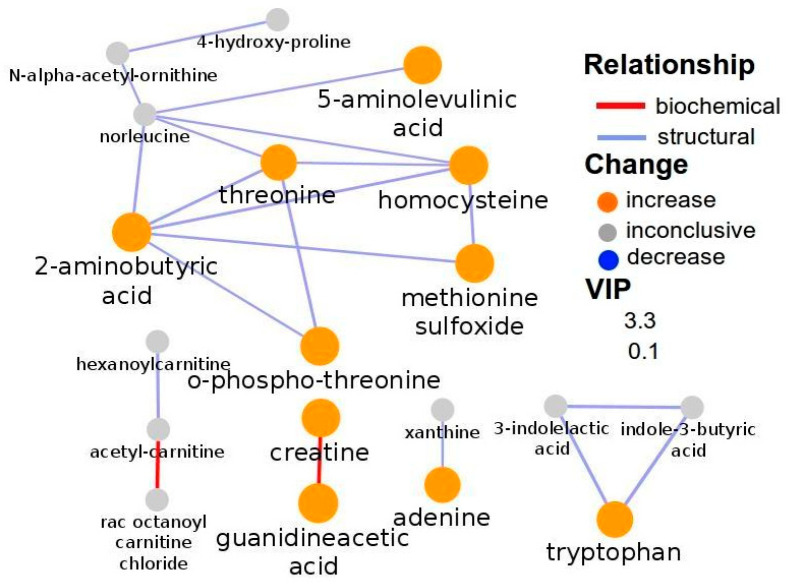
Biochemical network mapping of serum sample (ESI+ acquisition mode) of genotypes AA-AC. Metabolite connections are based on biochemical (red edges) and structural (violet edges) similarities. Structural similarity is shown for Tanimoto coefficients (≥0.7, solid edges) and relaxed scores. Node size displays model VIP and color the direction of change in AA-AC genotypes relative to CC genotype.

Network analysis was carried out using all metabolites significantly altered in the serum samples of both positive and negative (ESI+ and ESI−) modes of different genotypes of the SCZ susceptibility gene NOS1AP-rs12742393 (significant metabolites produced from the model are shown in Table 2 and Table 3). Metabolomics network analysis was performed for only substantial metabolites associated with genotype AA-AC of positive (ESI+) mode and genotype AA of negative (ESI−) mode since a variation of rs12742393 is associated with SCZ with an A allele as an SCZ risk allele [19]. The biochemical and structural similarity network for the positive (ESI+) acquisition mode of genotype AA-AC shows that these links are the metabolite relationships. Network mapping analysis illustrated a significant metabolic shift (Figure 3). In particular, based on the number of interconnected metabolites, the relationships indicated the strong association between 2-aminobutyric acids with threonine, homocysteine, methionine sulfoxide, and o-phospho-threonine. 2-Aminobutyric acid is an unnatural amino acid that is a key intermediate for synthesizing many important drugs. It can be produced by transaminase or dehydrogenase from α-ketobutyric acid, which can be synthesized enzymatically from the L-threonine.

**Figure 4:**
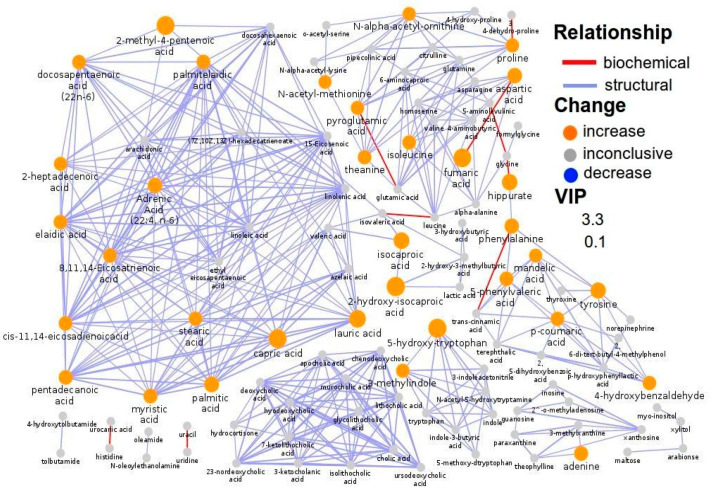
Biochemical network mapping of serum sample (ESI- acquisition mode) of genotypes AA. Metabolite connections are based on biochemical (red edges) and structural (violet edges) similarities. Structural similarity is shown for Tanimoto coefficients (≥0.7, solid edges) and relaxed scores (das. Node size displays model VIP and color the direction of change in AA genotype relative to AC and CC genotypes.

The biochemical and structural similarity network for the negative (ESI-) acquisition mode of genotype AA shows that these links are the metabolite relationships. Network mapping analysis illustrated a significant metabolic shift (Figure 4). In particular, fumaric acid is connected with aspartic acid; palmitic acid is connected with capric acid, lauric acid, adrenic acid, and elaidic acids; and 5-hydroxy-tryptophan is connected with 3-methylindole.

### 3.2. Enrichment Analysis Results

**Figure 5:**
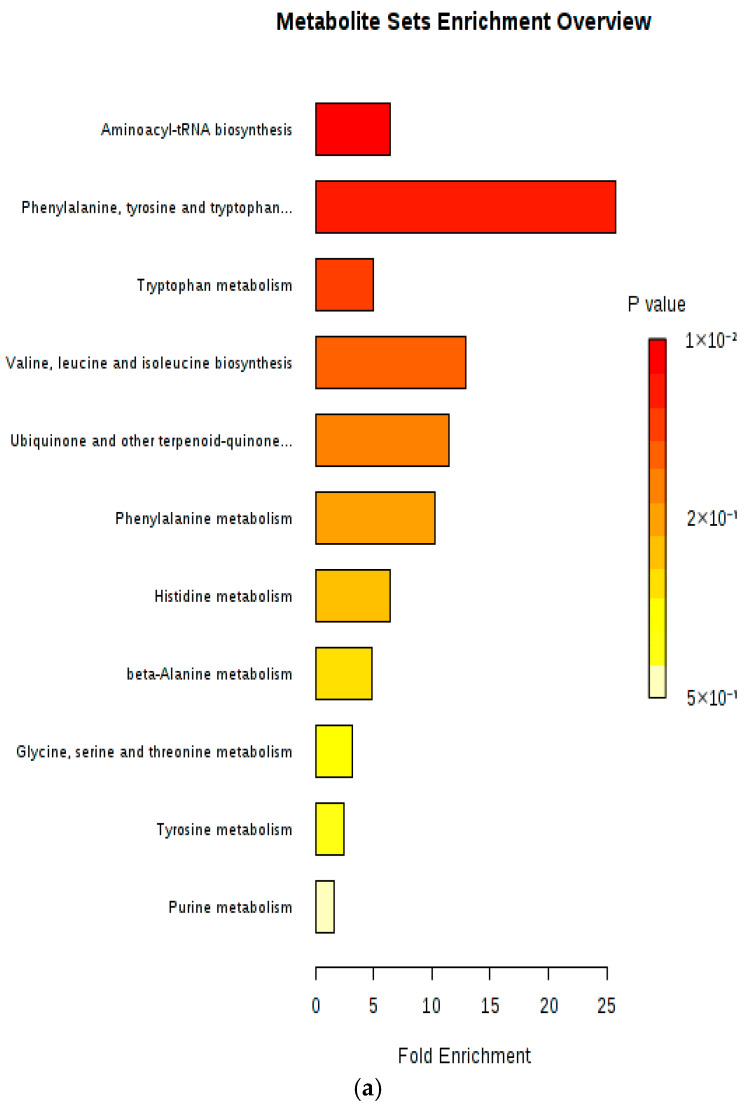

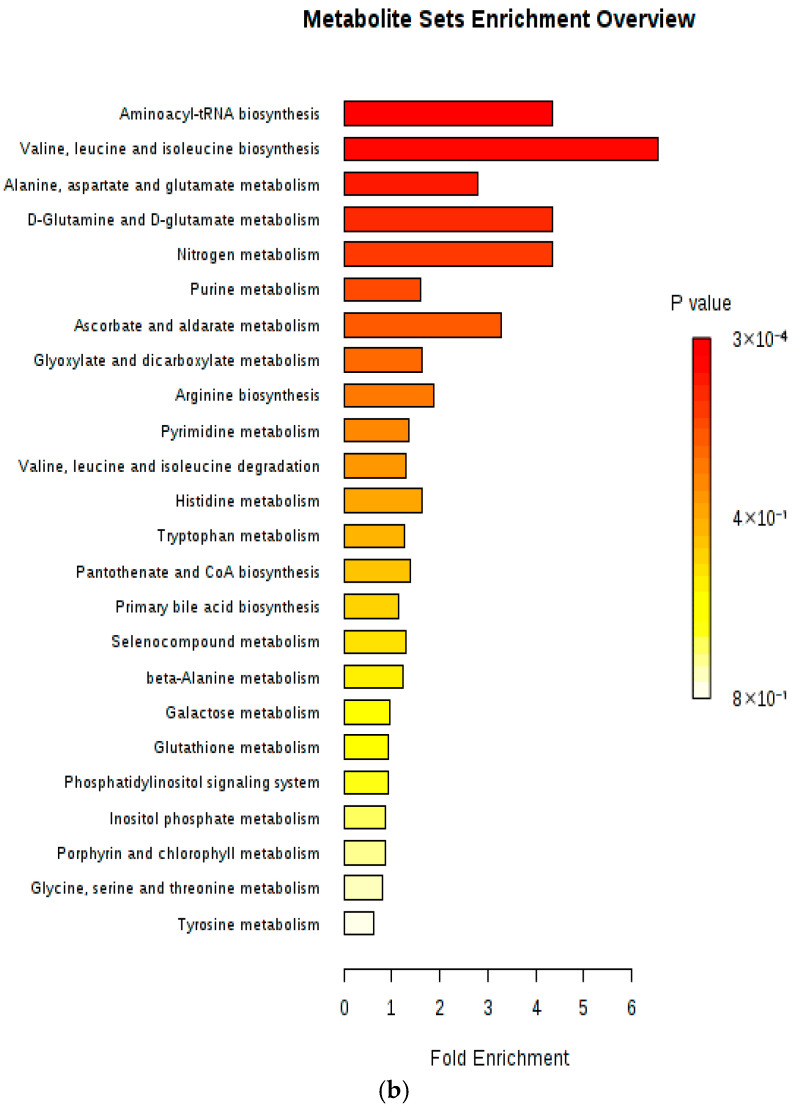
Summary plot of meaningful metabolic pathways in serum from the metabolite set enrichment analysis (MSEA), where metabolite sets are ranked according to Holm *p*-value, with hatched lines showing the cut-off of Holm *p*-value. The bar color is based on its *p*-value, and the bar length is fold enrichment. (**a**) Enrichment analysis of serum sample (UPLC, ESI+ acquisition mode) of genotypes AA-AC. Note that the second and fifth most important elements are phenylalanine, tyrosine, and tryptophan metabolism and ubiquinone; and other terpenoid–quinone metabolism. (**b**) Enrichment analysis of serum sample (UPLC, ESI− acquisition mode) of genotype AA.

Enrichment analysis was carried out using all metabolites significantly changed in the serum sample of positive and negative (ESI+ and ESI−) modes of different genotypes of the SCZ susceptibility gene NOS1AP-rs12742393. We found several important metabolic pathways by enrichment analysis shown in Figure 5. Aminoacyl-tRNA biosynthesis and tryptophan metabolism correlate in both genotype AA-AC and genotype AA, reflecting a strong association. We also found the enrichment analysis for metabolites randomly distributed in the genotypes AA-AC and genotype AA (Figure 5a) as well as those showing enrichment in genotype AA (Figure 5b). The enrichment analysis showed that the genotype AA provided information on metabolic pathways that are particularly relevant to the SCZ susceptibility gene NOS1AP [10,11].

## 4. Discussion

This study utilizes multivariate statistical analysis to identify key metabolites distinguishing AA-AC subjects from CC subjects in both positive and negative (ESI+ and ESI−) acquisition modes. Specifically, 16 metabolites were identified as significantly discriminating between AA-AC and CC subjects in the positive (ESI+) mode, while 40 metabolites were identified in the negative (ESI−) mode. Interestingly, tryptophan and 2-aminobutyric acid emerged as central metabolites in the metabolic network, exhibiting the highest node degree and betweenness centrality (Figure 3). Changes in L-tryptophan metabolism are particularly noteworthy, given its strong association with schizophrenia (SCZ). Additionally, palmitic acid and 5-hydroxy-tryptophan were found to have a strong interconnection, as evidenced by the number of interconnected metabolites (Figure 4). High betweenness centrality values (high influence on network flow and connectivity), often used to identify hub metabolites, are indicative of metabolites that play a pivotal role in controlling information flow within the network, with their removal potentially disrupting the communication between other nodes [24,25]. SCZ is rarely measured as a direct consequence of gene abnormalities; rather, it represents a perturbation of complex intracellular and intercellular networks that link tissues, organ systems, and the environment. Metabolomics, in combination with other “omics” platforms such as genomics, transcriptomics, proteomics, and metagenomics, holds significant promise for offering functional insights into complex diseases. However, integrating multi-omics data at a systems level remains a challenge. One common strategy is to analyze each omics dataset independently and then combine the results into a broader understanding, often through network-based approaches [26]. By integrating prior biological knowledge, such lists of significant features—such as metabolites, genes, and proteins—can be mapped onto knowledge-based networks, revealing important relationships between them and their associations with diseases or other phenotypes.

Previous studies have reported alterations in the levels of metabolites such as glutamine, arginine, histidine, ornithine, and phosphatidylcholine acyl-alkyl C38:6, suggesting their potential as candidate biomarkers for SCZ [27]. These findings suggest that metabolic pathways involving glutamine and arginine metabolism may reflect the genetic susceptibility of SCZ. Other research has highlighted the involvement of glucoregulatory processes and proline metabolism in SCZ [28]. More recently, a study indicated that SCZ patients at risk for metabolic syndrome exhibit specific metabolomic profiles [29].

Our pathway association analysis showed that metabolite sets associated with AA-AC and AA genotypes exhibited significant overlap with pathways implicated in SCZ, such as aminoacyl-tRNA biosynthesis and tryptophan metabolism. These results suggest that allele A of *NOS1AP* plays a significant role in SCZ susceptibility, with enriched metabolites potentially serving as early biomarkers for SCZ. Specifically, these metabolites could be critical for the identification of SCZ-associated alterations that may have been overlooked in previous studies.

The present findings point to substantial metabolic dysregulation in subjects with allele A of NOS1AP compared to those with allele C. Tryptophan was identified as a key metabolite in the metabolic network, with the highest node degree and betweenness centrality (Figure 3), highlighting its critical role in SCZ. Tryptophan, an essential amino acid and serotonin precursor, is known to be altered in several psychiatric disorders, including SCZ. The serotonergic pathway, which is crucial for regulating mood and cognition, may be impaired in SCZ patients, possibly due to enzyme blocks preventing the conversion of tryptophan to serotonin. Interestingly, although only a small fraction of tryptophan is converted into serotonin: the majority is metabolized into kynurenine. Additionally, the gut microbiome has been shown to influence tryptophan metabolism, contributing to an increase in indolyl compounds. Our metabolic network analysis further supports the involvement of tryptophan metabolism in SCZ, with significant enrichment in the tryptophan metabolism pathway (Figure 5b). There is currently no strong evidence directly linking 2-aminobutyric acid with SCZ in human or animal studies. Most reported associations in SCZ research pertain to GABA (γ-aminobutyric acid), glutamate, and kynurenine pathway metabolites [30].

Elevated levels of tryptophan and kynurenine are strongly associated with SCZ [31]. In the present study, both tryptophan and 2-aminobutyric acid exhibited high node degrees and betweenness centrality, reinforcing their importance in SCZ. Zhang et al. similarly found that SCZ risk allele A was associated with higher levels of tryptophan and kynurenine and lower serotonin levels [11]. Our findings corroborate this, showing that tryptophan is linked to various genotypes of SCZ in both positive and negative ion mode metabolic networks, with genotypes carrying allele A exhibiting a stronger effect than those with allele C.

Furthermore, the SCZ risk allele A exerts significant effects on metabolites within the serotonin and kynurenine pathway, as described by Zhang et al. Serotonin, a neurotransmitter involved in numerous physiological processes, has been implicated in various psychiatric disorders, including depression, Alzheimer’s disease, Parkinson’s disease, and SCZ [32,33,34,35,36]. As multi-omics methodologies evolve, the integration of genomic, transcriptomic, proteomic, and metabolomic data, coupled with systems biology and network medicine, promises to enhance our understanding of diseases like SCZ [26].

The utilization of network analysis in this study has enabled the identification of metabolites that significantly influence information flow and network connectivity. This approach offers a powerful tool for elucidating the complex biochemical alterations associated with SCZ and other pathological conditions. The future of network-based medicine holds great promise for uncovering new insights into disease mechanisms and developing targeted therapeutic strategies.

## 5. Conclusions

Biochemical and structural similarity networks, along with enrichment analysis of UPLC–QTOFMS positive and negative (ESI+ and ESI− mode)-based metabolomic profiling, suggest that the NOS1AP-rs12742393 gene plays a crucial role in a set of complex metabolic alterations, including changes in amino acids, organic compounds, fatty acids, and cholic acids. These findings highlight the potential of this approach in understanding the metabolic underpinnings associated with SCZ susceptibility. Furthermore, the network-based analysis provides insights into the specific metabolic signatures linked to different genotypes of NOS1AP-rs12742393, demonstrating its potential to contribute to a precision medicine approach for SCZ.

## Figures and Tables

**Table 1:** Demographic and clinical characteristics of the study population.

Characteristics	AA Genotype (N = 49)	AC Genotype (N = 49)	CC Genotype (N = 49)	*p*-Value
Age (mean years ± SD)	51.22 ± 14.12	50.51 ± 13.48	50.76 ± 13.87	0.96
Female, n (%)	26 (53.1)	26 (53.1)	27 (55.1)	
BMI (kg/m^2^, mean ± SD)	23.33 ± 3.58	23.64 ± 3.78	23.7 ± 4.72	0.90
Blood pressure (mean mm Hg ± SD)				
Systolic BP	120 ± 16	115 ± 16	114 ± 15	0.10
Diastolic BP	72 ± 18	72 ± 19	73 ± 16	0.97

**Table 2:** List of significant metabolites, obtained by multivariate statistical analysis, discriminating AA-AC subjects from CC subjects (ESI+ acquisition mode).

Metabolite	VIP Score	Trend in Genotypes AA-AC
[7-CD3]-1,3,7-Trimethyluric acid	1.38	down-regulated
Guanidineacetic acid	1.37	up-regulated
Hippurate	1.36	up-regulated
2-aminobutyric acid	1.32	down-regulated
Homocysteine	1.30	down-regulated
O-Phosphothreonine	1.30	up-regulated
Methionine sulfoxide	1.29	down-regulated
Creatine	1.27	down-regulated
5-aminolevulinic acid	1.25	down-regulated
Carnosine	1.25	up-regulated
Tryptophan	1.12	up-regulated
Threonine	1.10	up-regulated
Adenine	1.09	up-regulated
3,4-Dehydro-proline	1.08	up-regulated
4-aminophenol	1.05	up-regulated
Tyrosine	1.00	up-regulated

**Table 3:** List of significant metabolites, obtained by multivariate statistical analysis, discriminating AA subjects from AC and CC subjects. (ESI− acquisition mode).

Metabolite	VIP Score	Trend in Genotype AA
(S)-2-hydroxy-isocaproic acid	2.15	down-regulated
5-hydroxy-tryptophan	2.13	down-regulated
2-methyl-4-pentenoic acid	2.09	up-regulated
Capric acid (C10:0)	2.07	up-regulated
Fumaric acid	2.03	down-regulated
Lauric acid (C12:0)	1.83	up-regulated
Isocaproic acid	1.66	down-regulated
Hippurate	1.58	down-regulated
Tyrosine	1.55	down-regulated
Palmitic acid (C16:0)	1.48	up-regulated
Aspartic acid	1.41	down-regulated
Citric acid	1.40	down-regulated
Adrenic acid (cis-7,10,13,16-C22:4)	1.39	up-regulated
Phenylalanine	1.37	down-regulated
Allantoin	1.32	down-regulated
Myristic acid (C14:0)	1.29	up-regulated
Adenine	1.28	down-regulated
Pentadecanoic acid (C15:0)	1.27	up-regulated
5-phenylvaleric acid	1.23	up-regulated
Cis-8,11,14-eicosatrienoic acid (cis-8,11,14-C20:3, n-6)	1.23	up-regulated
Quinic acid	1.23	down-regulated
Palmitelaidic acid (trans-9-C16:1)	1.22	up-regulated
Eicosadienoic acid (cis-11,14-C20:2, n-6)	1.22	down-regulated
Oxypurinol	1.22	down-regulated
Docosapentaenoic acid (cis-4,7,10,13,16,C22:5, 22n-6)	1.21	up-regulated
Theanine	1.21	down-regulated
Isoleucine	1.20	down-regulated
P-coumaric acid	1.20	down-regulated
Stearic acid (C18:0)	1.19	up-regulated
Elaidic acid (trans-9-C18:1)	1.19	down-regulated
4-hydroxybenzaldehyde	1.18	down-regulated
3-methylindole	1.17	down-regulated
Proline	1.16	down-regulated
Pyroglutamic acid	1.14	down-regulated
1-methyluric acid	1.13	down-regulated
Heptadecanoic acid (C17:0)	1.12	down-regulated
N-alpha-acetyl-ornithine	1.11	up-regulated
Mandelic acid	1.09	down-regulated
N-acetyl-methionine	1.08	down-regulated
Taurine	1.07	down-regulated

## Data Availability

The dataset utilized in this study is available at the NIH Common Fund’s National Metabolomics Data Repository (NMDR) website.
